# Supplementary material for: Factors associated with COVID-19 among hospitalized patients with severe acute respiratory infections in Serbia, 2022–2023: A test negative case-control study
Source: PLoS One. 2024 Mar 18;19(3):e0299210. doi: 10.1371/journal.pone.0299210 (PMC10947665; doi:10.1371/journal.pone.0299210)
Supplement: Attachment — Submitted filename: New Microsoft Word Document (2).docx [file pone.0299210.s002.docx]

1. In introduction paragraph 2, reference 6 mentions about SARI surveillance and not about COVID vaccine effectiveness.
2. In paragraph 4, its mentioned as data was collected from to SARI sentinel sites in Serbia, but the data was collected from two hospitals.
3. In inclusion and exclusion criteria paragraph, its detailed as those who had a history of hospitalization within the 14 days of current admission were excluded—why?
4. Sample size calculation was not mentioned. Number of cases were 74 which is less for a common disease like COVID-19 or SARI.
5. For a case control study, cases to controls ratio should be a minimum of 1:1 to have adequate power. Here controls are less than the cases.
6. Study looks like a test negative case control study, but it is not mentioned in the article.
7. Test negative case control study can be used for assessing vaccine effectiveness, but not usually used for finding the risk factors of a disease.
8. Different types of statistical tests are used in the same table, but it is not mentioned in the foot note with labelling.
9. In results paragraph 2, it is mentioned that cases were significantly more in those using tobacco products, but, in table 1, it is mentioned as history of current smoking and not as usage of tobacco products. Is it used synonymously?
10. In results paragraph 3, the percentage of SARI patients who received a third dose of COVID vaccine was similar between cases and controls. However, in table 2 it is given as 32.4% vs. 58.3%.
11. In results paragraph 4, aren’t the controls took a median of one day more to seek medical help and not cases?
12. In table 4, the variable of primary COVID-19 vaccine series before hospitalization is not significant as the confidence interval includes unity.
13. In discussion paragraph 2, SARI patients were 64% less likely to be affected by COVID-19; this data was not mentioned in the results.
14. In discussion paragraph 3, seeking medical help is 42% lower among COVID-19 SARI patients than non COVID-19—not mentioned in results.
15. In discussion, inclusion of patients living in all parts of Serbia is described as the strength of the study, which is not well founded.
16. This study was not able to identify any new risk factors of COVID-19.
17. Abbreviations like ULRA, MLRA and MVRA are not universally used and better be avoided.
